# Rheumatoid nodules in thyroid gland parenchyma as an expression of rheumatoid arthritis: a case report

**DOI:** 10.1186/s13256-019-2113-9

**Published:** 2019-05-28

**Authors:** Efthimios Sivridis, Maria Kouroupi, Michael Ioannis Koukourakis, Stella Arelaki, Nikolaos Lyratzopoulos, Alexandra Giatromanolaki

**Affiliations:** 1 Department of Pathology, Democritus University of Thrace Medical School and University General Hospital of Alexandroupolis, 68100 Alexandroupolis, Greece; 2 Department of Radiotherapy/Oncology, Democritus University of Thrace Medical School and University General Hospital of Alexandroupolis, 68100 Alexandroupolis, Greece; 3 Department of Medicine, Democritus University of Thrace Medical School and University General Hospital of Alexandroupolis, 68100 Alexandroupolis, Greece

**Keywords:** Rheumatoid nodule, Thyroid gland, Rheumatoid arthritis

## Abstract

**Background:**

The rheumatoid nodule is the most common extra-articular manifestation of rheumatoid arthritis. When present, it is readily identified in conventional hematoxylin and eosin sections.

**Case presentation:**

We report a case with several rheumatoid nodules in a thyroid gland of a 33-year-old Greek woman with a 3-year history of rheumatoid arthritis treated with methotrexate, after having total thyroidectomy for hypothyroidism.

**Conclusion:**

To the best of our knowledge, this is the first time that rheumatoid nodules have been encountered in the thyroid gland.

## Background

Rheumatoid arthritis (RA) is a systemic inflammatory disease of an autoimmune nature, occurring in 0.5–1% of the population [1, 2]. Women are affected two to three times more commonly than men. The condition involves symmetrically the peripheral synovial joints – hands, feet, and wrists – particularly over bony prominences, leading to articular destruction and disability [1]. Extra-articular manifestations of RA occur in more than 35% of patients [3, 4] and are associated with severe active disease and a decreased survival rate [3]. Tissues that can be affected include extra-articular structures, such as tendons, ligaments, or fascia [3] and other organs, including skin [5], lungs [6], oral mucosa [7, 8], gastrointestinal tract [8], and cardiovascular [9] and neurological systems [10].

In all extra-articular sites, the characteristic pathological finding has been the rheumatoid nodule – solitary or multiple nodules, 0.2 to 5 cm or more in diameter – containing areas of fibrinoid necrosis surrounded by palisading histiocytes [6, 11]. To the best of our knowledge, such nodules have not previously been described in the thyroid gland parenchyma.

## Case presentation

A 33-year-old Greek woman was found to have hypothyroidism following a thorough investigation of migraines, after a road traffic accident. The event was complicated with craniocerebral injury necessitating tracheostomy. Her past medical history included RA of 3-year duration treated with methotrexate (2.5 mg three times per day), and topiramate medication for migraines (200 mg twice a day). On clinical examination, the thyroid gland was painless and not palpable. Laboratory tests confirmed a positive rheumatoid factor (RF) with normal antithyroglobulin (anti-TG) and thyroid peroxidase antibodies (anti-TPO) (16 U/ml and 16.7 U/ml, respectively). An ultrasound-guided fine needle aspiration biopsy performed in a private clinic showed distinct nodules in the lower pole of the left thyroid lobe, which were reported as being suggestive, though not conclusive, of malignancy (category V Bethesda) [12]. She was put on thyroxine (T4) treatment and when she became euthyroid with thyroid-stimulating hormone (TSH) of 0.89 μIU/ml, triiodothyronine (T3) of 1.30 ng/mL, and T4 of 7.2 μg/dl, she was subjected to a total thyroidectomy in our hospital.

**Fig. 1:**
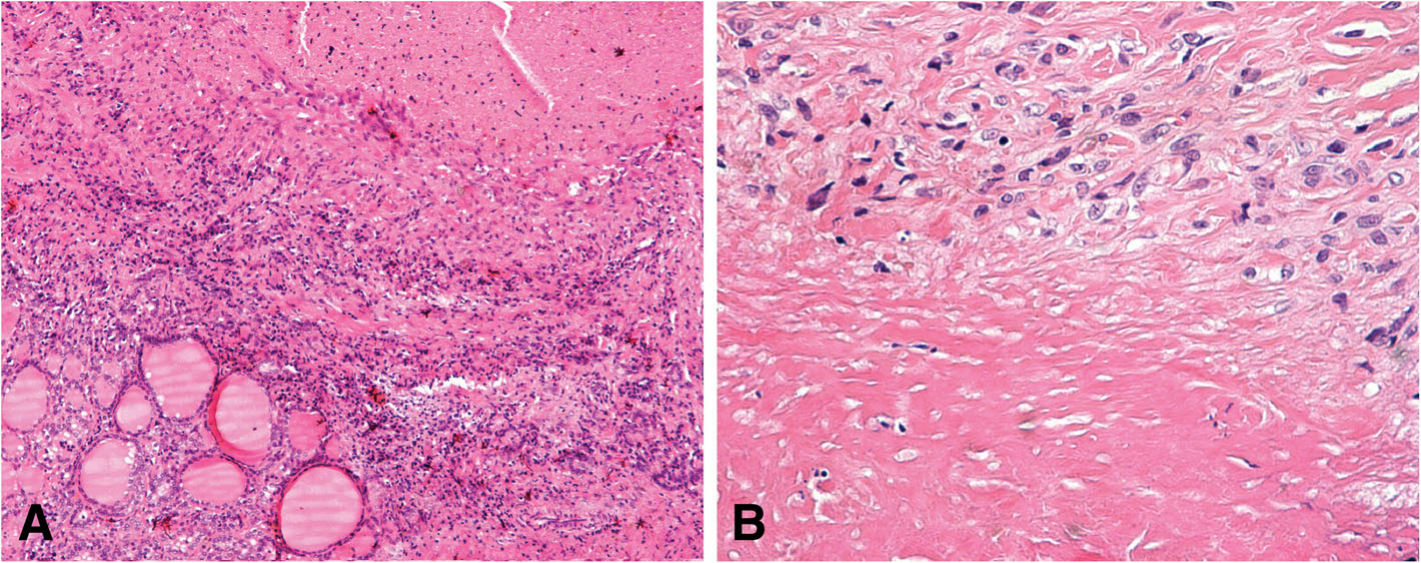
Rheumatoid nodule **a** x10 magnification, **b** x40 magnification

The resected thyroid specimen, received in three pieces (4 × 3 × 1.5 cm; 4.5 × 2.7 × 1 cm; and 5 × 2.5 × 1 cm), was surrounded by multiple adhesions; its total weight was 36 g. Two of the specimens exposed a cut surface composed of clusters of small irregular follicles separated by reticular connective tissue, while the gland architecture of the third specimen (5 × 2.5 × 1 cm) was replaced in part by five small areas of amorphous necrotic tissue. On microscopic examination the necrotizing lesions (0.2 to 0.4 cm in greatest diameter) corresponded to rheumatoid nodules, composed of a central area of fibrinoid necrosis surrounded by palisading histiocytes; these, in turn, were encircled by fibroblasts, lymphocytes, and plasma cells (Fig. 1). There was a proliferation of small blood vessels around the nodule, lacking any perivascular inflammation. Yet, the surrounding thyroid tissue showed focal lymphocytic thyroiditis, with typical germinal centers, alternating with stromal fibrosis. No evidence of infection, sclerosing thyroiditis, thyroid carcinoma, or lymphoma was noted.

## Discussion and conclusions

The rheumatoid nodule is the hallmark of extra-articular manifestation of RA. The lesion is usually asymptomatic and, as indicated in this and in previously described cases, it is associated with seropositive cases (positive RF) and methotrexate treatment [6, 13, 14]. It has been repeatedly reported that an interruption to methotrexate medication leads to a decrease in the size of the rheumatoid nodules [15–17]. Other features, which characterized our case, were the average levels of antithyroid antibodies, the lack of clinical symptoms, and the presence of typical rheumatoid nodules with central fibrinoid necrosis and palisading histiocytes at the periphery. Such nodules are commonly encountered in synovial joints (destructive polyarthritis) and extra-articular organs including skin and subcutaneous tissues [5, 18], heart [19], lungs [6], kidneys [20], nervous system [10], and gastrointestinal system [8].

Common extra-articular manifestations include anemia (61%), thrombocytosis (16%), and pulmonary involvement (10%), while renal amyloidosis, vasculitis, and Felty syndrome occur less frequently (6%, 2%, and 1%, respectively) [20]. Skin manifestations are probably the result of small vessel vasculitis. They are often associated with hemorrhages, ulcers, digital gangrene, and pyoderma gangrenosum [5]. Pulmonary manifestations in RA are rather frequent. In fact, autopsy studies reported pleural effusions in 50% of cases, although only a small proportion of them are clinically detected [21].

Furthermore, the disease is frequently associated with interstitial lung disease [22]. Oral manifestations include dryness and swelling of salivary glands and often Sjögren’s syndrome [8]. Gastrointestinal complications in RA have been reported as being mainly drug-induced. Primary involvement of the gastrointestinal tract may also occur in the form of mesenteric vasculitis causing intestinal infarction but is extremely rare [8]. There is also an increased risk of cardiovascular disease [9, 19], with the risk for myocardial infarction in female patients with RA being twice to three times higher than that of women without RA [9]. Anemia is, by far, one of the most common extra-articular manifestations of RA [23]. The cause of anemia in the case of RA is multifactorial: it is mainly caused by medications, gastrointestinal hemorrhage, or bone marrow suppression. Neurological manifestations in the form of peripheral neuropathy are not uncommon in patients with RA [10]. The underlying mechanism is small vessel vasculitis of the vasa vasorum of the nerves leading to ischemic neuropathy and demyelination. Cerebral strokes are common.

Rheumatoid nodules or endocrine manifestations have not been reported in endocrine organs in patients with RA. There has been, of course, an unusual case of active RA with a rheumatoid nodule developing at the thyroid bed after total thyroidectomy for Hashimoto’s thyroiditis [24]. Note that the previously resected thyroid parenchyma was free of rheumatoid nodules. It is interesting, however, that while Hashimoto’s autoimmune thyroiditis is usually accompanied by the presence of thyroid peroxidase (TPO) and thyroglobulin (TG) antibodies, focal lymphocytic thyroiditis is not; it may, however, represent an early subclinical form of autoimmune thyroiditis [25]. It appears, therefore, that rheumatoid nodules can develop independently of a thyroid background.

RA is a chronic inflammatory disease of an autoimmune nature characterized by articular involvement, often in the presence of RF and rheumatoid nodules. Although considered a joint disease, RA is not infrequently associated with extra-articular involvement. Yet, the reported case is the first described in endocrine gland parenchyma and was free of symptoms. Extra-articular RA is, in general, a severe condition, usually associated with an increased mortality rate. Early recognition and treatment are essential to the patients’ welfare.

## Abbreviations

anti-TG: Antithyroglobulin
anti-TPO: Thyroid peroxidase antibodies
RA: Rheumatoid arthritis
RF: Rheumatoid factor
T3: Triiodothyronine
T4: Thyroxine
TG: Thyroglobulin
TPO: Thyroid peroxidase
TSH: Thyroid-stimulating hormone
